# Microglia and macrophage metabolism: a regulator of cerebral gliomas

**DOI:** 10.1186/s13578-024-01231-7

**Published:** 2024-04-17

**Authors:** Yue Deng, Qinyan Chen, Chao Wan, Yajie Sun, Fang Huang, Yan Hu, Kunyu Yang

**Affiliations:** 1 Cancer Center, Union Hospital, Tongji Medical College, Huazhong University of Science and Technology, Wuhan, 430022 China; 2 Hubei Key Laboratory of Precision Radiation Oncology, Wuhan, 430022 China; 3 Institute of Radiation Oncology, Union Hospital, Tongji Medical College, Huazhong University of Science and Technology, Wuhan, 430022 China

**Keywords:** Glioma, Glioma-associated macrophages, Metabolism, Targeted therapy

## Abstract

Reciprocal interactions between the tumor microenvironment (TME) and cancer cells play important roles in tumorigenesis and progression of glioma. Glioma-associated macrophages (GAMs), either of peripheral origin or representing brain-intrinsic microglia, are the majority population of infiltrating immune cells in glioma. GAMs, usually classified into M1 and M2 phenotypes, have remarkable plasticity and regulate tumor progression through different metabolic pathways. Recently, research efforts have increasingly focused on GAMs metabolism as potential targets for glioma therapy. This review aims to delineate the metabolic characteristics of GAMs within the TME and provide a summary of current therapeutic strategies targeting GAMs metabolism in glioma. The goal is to provide novel insights and therapeutic pathways for glioma by highlighting the significance of GAMs metabolism.

## Introduction

Glioma is one of the most common primary brain tumors, accounting for 25% of central nervous system (CNS) tumors [1]. It typically arises from primary neural stem or glial cells, forming a complex network of neurons, astrocytes, resident myeloid cells, and microglia [2]. The current standard treatment for gliomas is surgical resection coupled with adjuvant radiotherapy and chemotherapy, but survival rates for glioma patients remain low due to their high aggressiveness and therapy resistance [3, 4]. According to the 2007 World Health Organization (WHO) classification criteria for gliomas, glioblastoma (GBM) is classified as stage IV [5], with a median survival of only 8–18 months [1, 3]. There is an urgent need for more effective and less toxic treatment strategies to improve patient survival.

In recent years, it has been gradually recognized that immune cells’ responsiveness to tumors depends mainly on their metabolic program, which is related to the type and function of immune cells [6]. Tumor cells undergo metabolic reprogramming to obtain unlimited proliferation capacity and evade immune surveillance [7]. The rewired metabolic program of tumors creates a hypoxic, acidic, and nutrient-deficient tumor microenvironment (TME), resulting in immune cells which are immunosuppressive and promoting tumor progression. This becomes a huge barrier to anti-tumor immunity [8]. Therefore, targeted metabolic therapy has the potential not only to hinder tumor growth but also to remodel TME and activate anti-tumor immune responses, and enhancing the efficacy of immunotherapy. Thus, metabolic therapy provides a new therapeutic direction for improving prognosis of glioma patients [9, 10].

Due to the existence of blood–brain barrier, the brain is considered as a unique immune organ [11]. Microglia and tumor-associated macrophages/monocytes, also known as glioma-associated macrophages (GAMs), are the predominant immune cells that extensively infiltrate in the glioma microenvironment, constituting approximately 30% of the tumor mass [12–14]. Numerous studies demonstrate that glioblastoma cells stimulate GAMs to produce immunosuppressive cytokines, which in turn promote tumor growth, facilitate regulatory T lymphocytes (Treg) recruitment, and induce T cell apoptosis to suppress anti-tumor immune responses [15, 16]. Given their importance as immune cells in glioma, targeting GAMs through metabolic therapy may emerge as a highly promising approach. This review provides an overview of metabolic studies focusing on GAMs, aiming to present innovative ideas for glioma treatment.

## Metabolic profiles of macrophages in brain

**Fig. 1:**
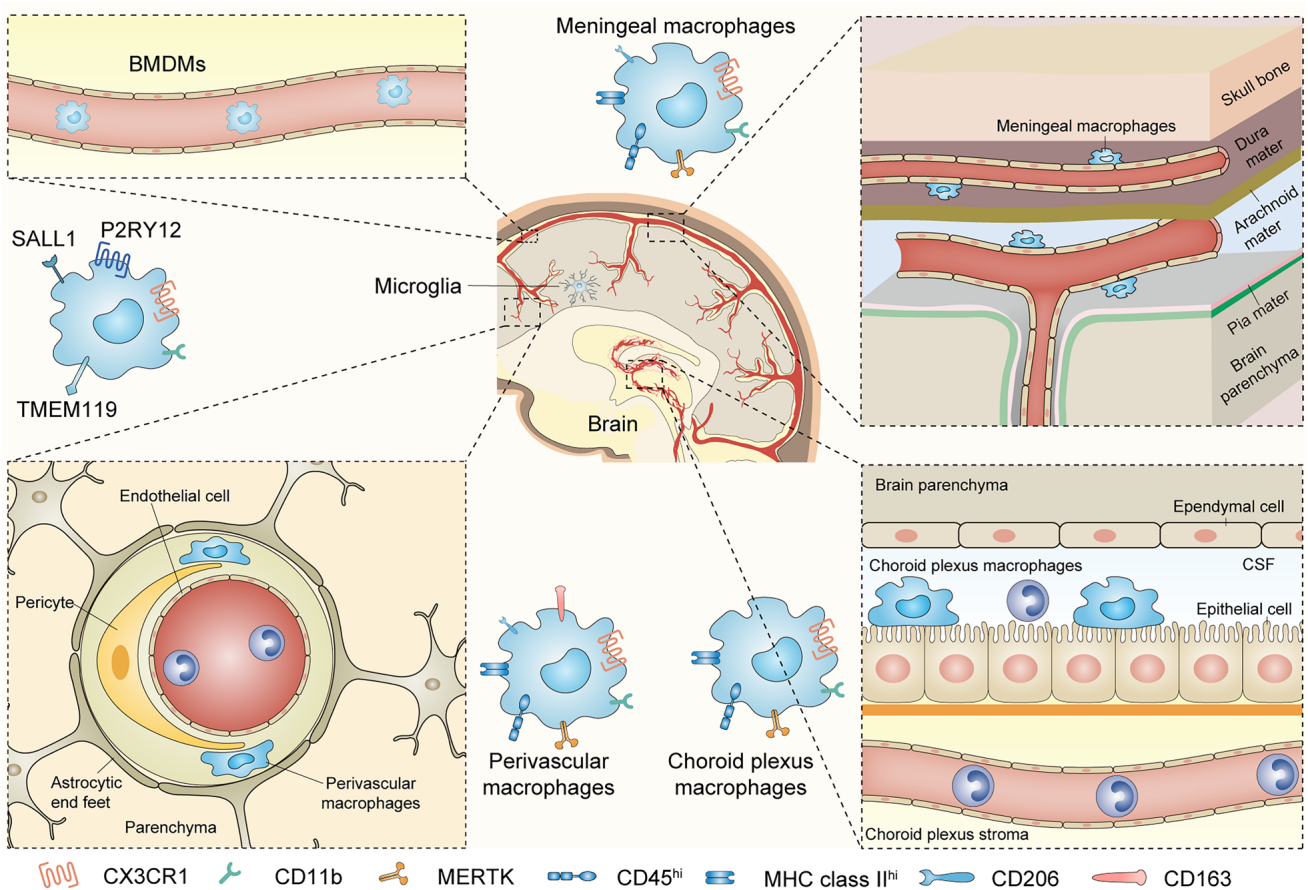
Classification of brain macrophages. Brain macrophages are typically categorized into three major types. Microglia are localized in the central nervous system parenchyma and specifically express P2Y purinoceptor 12 (P2RY12), transmembrane protein 119 (TMEM119) and Sal-like protein 1 (SALL1). The other group is perivascular, meningeal and choroid plexus macrophages located at the interface between the parenchyma and the circulation, which are less well defined because many markers are expressed overlapping. BMDMs are distributed in the extensive network of vasculature. CX3CR1, CX3C-chemokine receptor 1; CSF, cerebrospinal fluid; BMDMs, bone marrow-derived macrophages

Brain macrophages are typically categorized into the following three types (Fig. 1). Microglia are originated from embryonic yolk sac precursors and localized in the central nervous system parenchyma, account for approximately 5–10% of total brain cells [13, 17, 18]. Perivascular, meningeal and choroid plexus macrophages are located at the interface between parenchyma and circulation. Perivascular and meningeal macrophages arise from embryonic yolk sac precursors, whereas choroid plexus macrophages have both embryonic and adult hematopoietic origin [19, 20]. The central nervous system also contains an extensive network of vasculature with circulating myeloid cells such as monocytes, granulocytes, and dendritic cells [21].

**Fig. 2:**
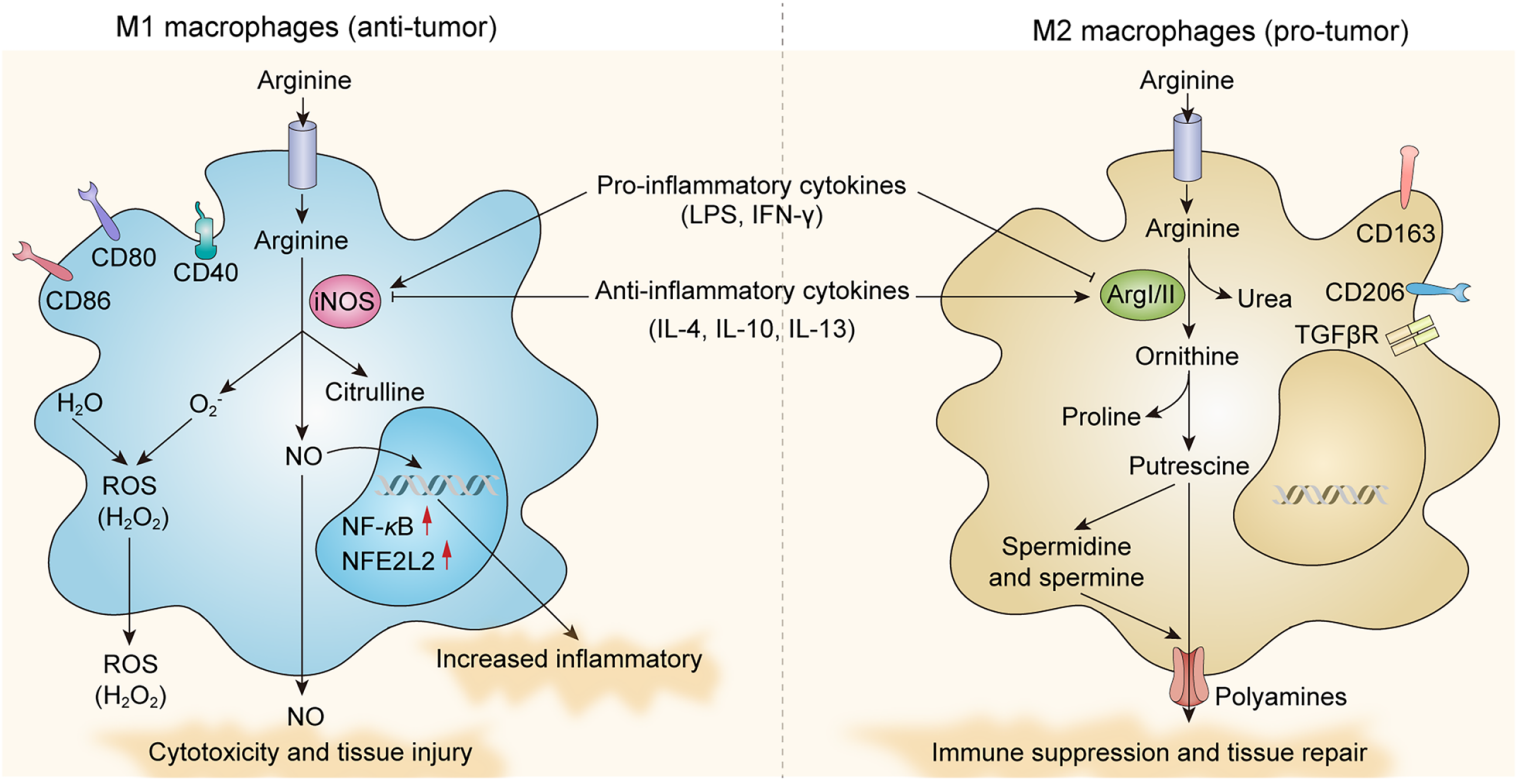
Features of arginine metabolism in M1-type and M2-type macrophages. M1 macrophages are induced by pro-inflammatory cytokines or LPS and are characterized by the expression of iNOS to produce immune response modulator NO and citrulline. M2 macrophages are induced by anti-inflammatory cytokines such as IL-4, IL-10, and IL-13 and express Arg1 to generate L-ornithine and urea, which are precursors of polyamine and proline synthesis. Functionally, M1 macrophages have powerful antitumor activity, while M2 macrophages promote tissue repair, suppress inflammation and promote tumor development. iNOS, inducible nitric oxide synthase; NO, nitric oxide; ROS, reactive oxygen species; NFE2L2, gene coding for the nuclear factor erythroid 2-like 2 (Nrf2); NF-κB, gene coding for the nuclear factor kappa-b subunit; LPS, lipopolysaccharide; IFNγ, interferon γ; IL-4, interleukin-4; IL-10, interleukin-10; IL-13, interleukin-13; Arg I/II, arginase type I/II; TGFβR, transforming growth factor beta receptor

According to the function of macrophages, they are mainly divided into two types, the classical activated or inflammatory macrophages (M1 macrophages) and the alternatively activated or anti-inflammatory macrophages (M2 macrophages) [22, 23] (Fig. 2). Macrophages exhibit remarkable plasticity and when the tissue microenvironment changes, they will functionally respond to different signals in the environment and transform from one phenotype to another, which is known as macrophage polarization [23–26]. M1 macrophages are typically induced by Th1 cytokines or bacterial lipopolysaccharide (LPS) with markers including CD40, CD80, CD86, TLR2, TLR4 and MHC-II [27]. Pro-inflammatory cytokines such as TNF-α, IL-1α, IL-1β, IL-6, and IL-12, as well as Th1 cell-attracting chemokines such as CXCL9 and CXCL10 are usually secreted [23, 24, 28]. M1 macrophages mediate tissue damage by activating the nicotinamide adenine phosphate dinucleotide (NADPH) oxidase system to generate reactive oxygen species (ROS) [26]. To prevent excessive tissue damage caused by M1 macrophages, the M2 macrophages inhibit the formation of chronic inflammation in a regulating mechanism. Th2 inflammatory factors such as IL-4, IL-10, and IL-13 generally drive M2-type polarization by activating STAT6 or STAT3 [29, 30]. M2 macrophages characteristically express CD163, CD206, scavenging, mannose and galactose receptors [27]. And they secrete cytokines such as IL-10, TGF-β, VEGF, and chemokines such as CCL17, CCL22, and CCL24 that promote Th2 cells recruitment [28, 31]. Moreover, macrophages with different polarization states usually express different metabolic biomarkers. M1 macrophages are characterized by the expression of inducible nitric oxide synthase (iNOS), which produces immune response modulator nitric oxide (NO) and citrulline. M2 macrophages express a high level of arginase 1 (Arg1) to generate L-ornithine and urea, which are precursors of polyamine and proline synthesis contributing to tumor proliferation and progression [26, 32–35].

In tumor progression, M1 macrophages have powerful anti-tumor activity. On the one hand, significant activation of the Toll-like receptor signaling pathway facilitates the clearance of pathogens and tumor cells [36, 37]. On the other hand, highly expressed antigen-presenting MHC complexes accelerate the activation of acquired immune responses [38–40]. Moreover, M1 macrophages can directly act on target cells by producing NO and ROS [41]. Meanwhile, they secrete cytokines to promote inflammatory responses and recruit anti-tumor T-cell infiltration [42]. However, M2 macrophages participate in Th2 responses, have strong phagocytic capacity and are involved in clearance of cellular fragments and apoptotic cells. They play a crucial role in promoting tissue repair, wound healing, angiogenesis, and fibrosis. Thus, M2 macrophages not only suppress inflammation but also facilitate angiogenesis, immune suppression, tumor formation, and development [25, 43–46].

Macrophages with different polarization states have distinct metabolic profiles [27, 34]. It has been shown that both M1 and M2 macrophages can significantly regulate glucose and amino acid metabolism. Since LPS and Th1 cytokine-activated M1 macrophages are often associated with acute infections and are required to rapidly trigger strong antimicrobial activity, their metabolic pathway is remodeled from oxidative phosphorylation to glycolysis to adapt to hypoxic environment [33, 47–49]. In contrast, since M2 macrophages play a crucial role in wound healing and tissue repair, they require glucose oxidation and fatty acid oxidation to provide a constant supply of energy, hence fatty acid oxidation is the most important metabolic pathway for M2 macrophages [27, 33, 43, 44]. In addition, to fit into the distinctive tumor microenvironment, macrophages tend to undergo metabolic reprogramming as well. For example, during migration, macrophages need to adjust to the hypoxic environment by converting their metabolic pathway to glycolysis due to the difference in oxygen concentration in different tissues. Activation of hypoxia-inducible factor (HIF)-1 and -2 triggers functional changes in macrophages, including expression of chemokines and chemokine receptors, such as CXC chemokine receptor 4 (CXCR4), CXCL12, angiogenic factor and vascular endothelial growth factor (VEGF) [27, 35]. In this way, macrophages assist in coordinating tissue adaptation to oxygen gradients and hypoxic conditions.

**Fig. 3:**
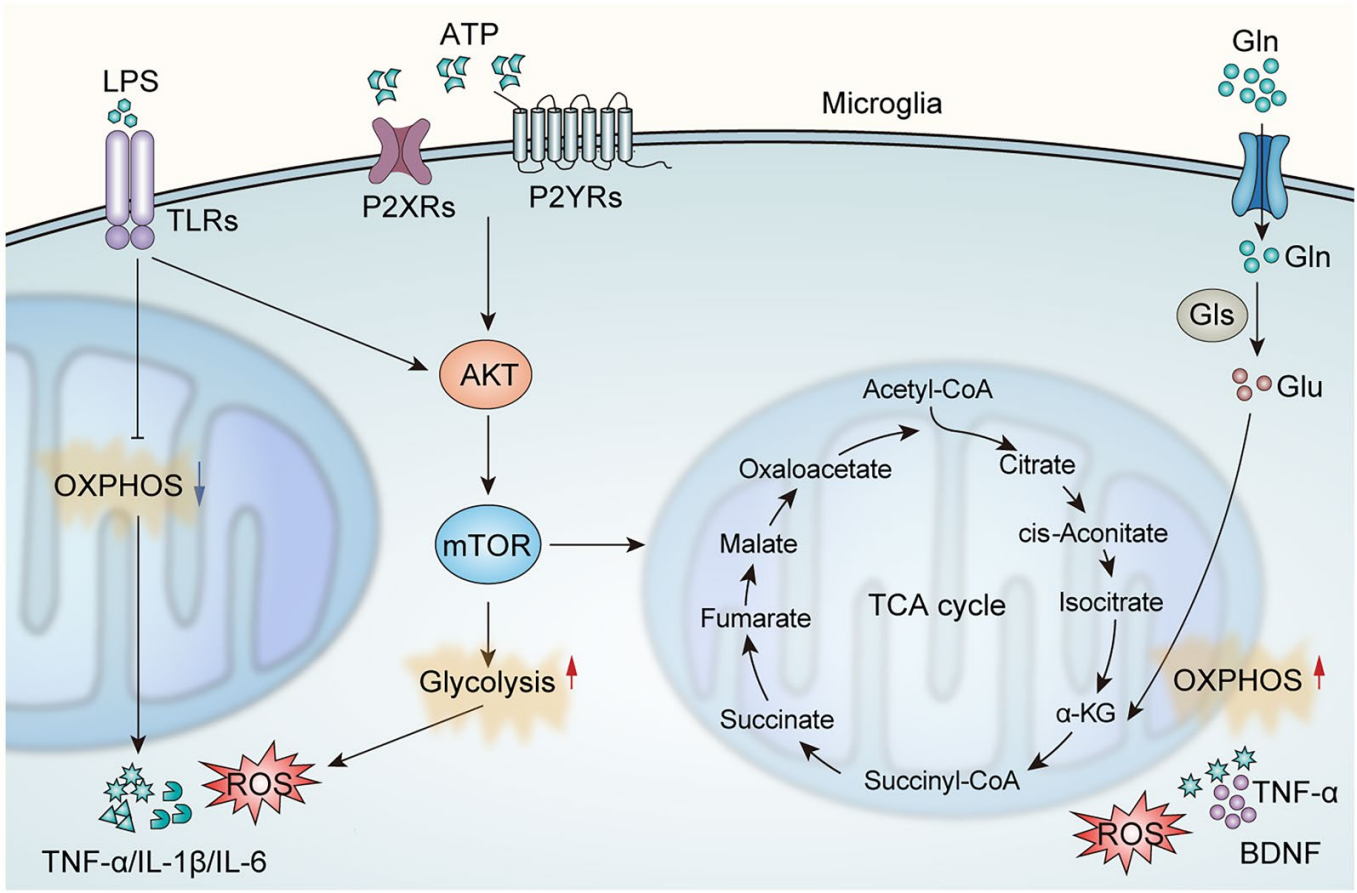
Metabolic characteristics of microglia. LPS and ATP effectively promoted glycolysis via the mTOR signaling pathway in microglia, while LPS inhibited OXPHOS but ATP activated OXPHOS. Microglia can also utilize glutamine as an energy source to maintain mitochondrial activity. LPS, lipopolysaccharide; TLRs, Toll-like receptors; OXPHOS, oxidative phosphorylation; ROS, Reactive oxygen species; TNF-α, tumoral necrosis factor α; IL-1β, interleukin-1β; IL-6, interleukin-6; mTOR, mammalian target of rapamycin; Gln, Glutamine; Glu, Glutamate; Gls, Glutaminase; BDNF, brain-derived neurotrophic factor

Numerous studies have focused on profiling the metabolic characteristics of microglia in gliomas (Fig. 3). Hu et al. monitored the metabolic profiles of LPS and IL-4 activated microglia by hippocampal analyzer and found that LPS and ATP effectively promoted glycolysis in microglia, while LPS inhibited oxidative phosphorylation but ATP activated oxidative phosphorylation [50]. Whereas, changes in glycolysis and oxidative phosphorylation were not apparent in IL-4-activated microglia. Mechanistically, the mTOR signaling pathway can effectively regulate the different metabolic reprogramming mediated by LPS and ATP. Blockade of mTOR or glycolysis metabolism can effectively reduce microglia-mediated neuroinflammation in multiple disorders, indicating an essential role of the mTOR pathway in microglia function and revealing an important contribution of microglia metabolic programming to immune regulation.

In addition, Bernier et al. found that microglia are metabolically mobile and proceed with immunosurveillance functions powered by glutamine even without glucose [51]. The results showed that microglia were significantly more metabolically active under the condition of glucose and/or glutamine and maintained mitochondrial activity in the presence of glutamine alone, indicating that microglia can utilize glutamine as an energy source. It was further revealed that the metabolic conversion process of microglia is dependent on the mTOR signaling pathway, resulting in a rapid switch between glycolysis and glutamine catabolism, and this metabolic plasticity enables microglia to maintain their critical immunosurveillance and phagocytosis despite the dysregulation of brain nerve energy homeostasis.

## Metabolic interactions between tumor cells and glioma-associated macrophages

**Fig. 4:**
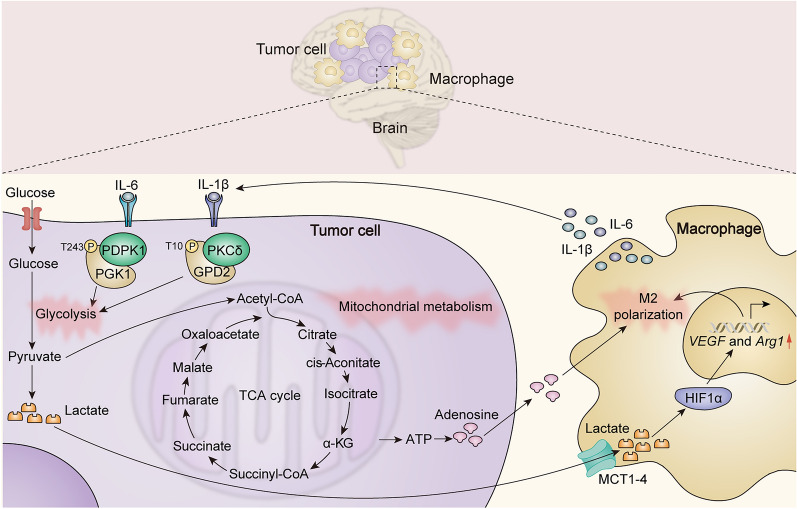
Metabolic interactions between tumor cells and GAMs. Tumor cells cause massive accumulation of lactate in the TME via the Warburg effect, and lactate further induces macrophages to express VEGF and Arg1 via the HIF1α signaling pathway, which promotes polarization of macrophages toward M2 phenotype. Adenosine produced by tumor cell metabolism can also promote M2-type polarization in macrophages. Macrophages release IL‐1β, which triggers phosphorylation of GPD2 at T10 via PKCδ, to promote glycolysis in glioma cells. Macrophages produce IL-6, which induces PDPK1-dependent PGK1 T243 phosphorylation to promote glycolysis in glioma cells. PDPK1, 3-phosphoinositide-dependent protein kinase 1; PGK1, phosphoglycerate kinase 1; PKCδ, protein kinase-delta; GPD2, glycerol‐3‐phosphate dehydrogenase; IL-1β, interleukin-1β; IL-6, interleukin-6; HIF1α, hypoxia inducible factor 1 alpha; MCT1-4, monocarboxylate transporter1-4; VEGF, vascular endothelial growth factor; Arg1, arginase 1

With the progression of tumor, the polarization states and function of GAMs evolve with progression of glioma. At the initial stage of tumorigenesis, GAMs have strong immunological activity. However, during the late stage of neoplastic progression, with the increment of cytokines such as IL-4, IL-10 and TGF-β in the TME, GAMs are polarized into M2 phenotype [52–54], which promotes tumor growth, angiogenesis and exacerbates the energy metabolism disorder, thus leading to tumor progression and treatment resistance [53, 55, 56] (Fig. 4).

### GAMs metabolic remodeling by the tumor microenvironment

Several metabolites produced by tumor cells in the TME, such as lactate, tryptophan and adenosine, also functionally remodel GAMs. Generally, even in the presence of oxygen and fully functional mitochondria, tumor cells are supplied with energy via the glycolytic pathway, which phenomenon is known as the Warburg effect, and consequently leads to a massive accumulation of lactate in the TME [9, 10]. It has been extensively shown that lactate, while facilitating tumor invasion, metastasis and angiogenesis, also exerts immunosuppressive effects, impairing antigen presentation of dendritic cells (DCs), inhibiting cytotoxicity of CD8^+^ T cells, and inducing apoptosis of NK cells [57]. The remodeling of GAMs by lactate is manifested in the induction of VEGF and Arg1 expression via the HIF1-α signaling pathway, which promotes the polarization of GAMs toward M2 and assists GAMs in their pro-tumor function.

Acidity is another characteristic of the tumor microenvironment. Local hypoxia and glycolysis induced by the Warburg effect render tumor cells in an acidic state, whereas acidosis is detrimental to cell growth. Therefore, metabolically generated protons must be expelled from the tumor cytosol to maintain cellular function, and this process is achieved through Na/H exchanger isomer 1 (NHE1). NHE1 drives H^+^ efflux along with Na^+^ influx to maintain the pH in tumor cells at 7.3–7.5, which can provide a driving force for tumor cells and ensure the normal maintenance of glycolytic [58]. However, in the case of immune cells, the acidotic environment of TME suppresses the function of immune cells and promotes the polarization of GAMs to M2 type [58–60].

Furthermore, combined with metabolomics and gene expression profiling, Pravin et al. performed a centralized and comprehensive analysis comparing 80 patients with GBM and 28 patients with low-grade astrocytomas [61]. It was proved that tryptophan and adenosine metabolism led to the accumulation of Treg and M2 macrophages, respectively, in the microenvironment of GBM patients and was validated in the mouse model, suggesting that metabolic reprogramming may possibly underlie an important factor causing immune tolerance in GBM.

### Effects of GAMs on tumor cell metabolism

GAMs are capable of secreting a wide range of chemokines or cytokines that modulate tumor cell metabolism. Chemokines such as CCL5 and CCL18 produced by GAMs could upregulate the key enzymes of tumor cell glycolytic pathway, such as lactate dehydrogenase (LDH) and glucose-6-phosphate dehydrogenase (G6PD), further promoting tumor cell glycolysis and exacerbating the accumulation of lactate in the microenvironment, forming a vicious cycle [62]. Furthermore, IL-1β, produced by M2 macrophages, activates the phosphorylation of glycerol-3-phosphate dehydrogenase (GPD2) on threonine 10 (GPD2 pT10) in glioma cells, which enhances the affinity of GPD2 pT10 to substrate, and improves the catalytic rate of glycolysis in glioma cells [63]. On the other hand, IL-6, secreted by M2 macrophages, enhances the phosphorylation of phosphoglycerate kinase 1 (PGK1) threonine 243 mediated by 3-phosphatidylinositol-dependent protein kinase 1 (PDPK1) in tumor cells, thereby regulating the direction of PGK1-catalyzed reactions and promoting tumor cell glycolysis and tumorigenesis [64].

## Therapeutic strategies targeting metabolism between GAMs and Gliomas

**Fig. 5:**
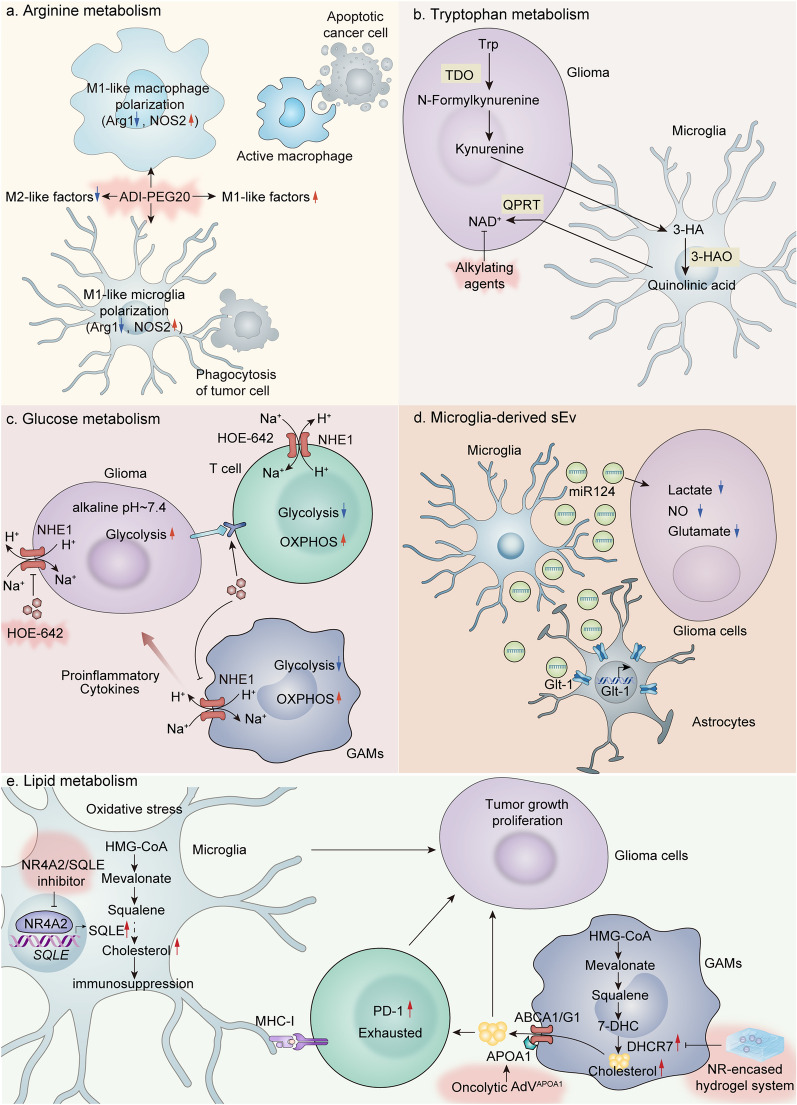
Therapeutic strategies targeting metabolism between GAMs and Gliomas. **a** Therapeutic strategies targeting arginine metabolism. ADI-PEG20, an arginine consumer, decomposes arginine in the TME, and induces upregulation of NOS2 expression and downregulation of Arg1 to promote M1-like polarization. In addition, ADI-PEG20 enhances the phagocytic ability of GAMs, and promotes the M1-like factors secretion. **b** Therapeutic strategies targeting tryptophan metabolism. Endogenous tryptophan metabolites and the NAD^+^ precursor quinolinic acid render gliomas resistant to radiotherapy and chemotherapy. Alkylating agents may alleviate therapeutic resistance by depleting intracellular NAD^+^. **c** Therapeutic strategies targeting glucose metabolism. HOE-642, an inhibitor of NHE1, significantly promotes infiltration of GAMs and T cells, upregulates mitochondrial OXPHOS pathway genes, enhances mitochondrial activity in immune cells, and diminishes glycolysis in GAMs and T cells. **d** Therapeutic strategies using microglia-derived sEV. Microglia-derived sEV carrying miR-124 alters the metabolism of glioma cells and reduces the release of lactate, NO and glutamate. In addition, sEV increases the expression of Glu transporter Glt-1 in astrocytes. **e** Therapeutic strategies targeting lipid metabolism. NR4A2 or SQLE inhibitors suppress tumor growth and reversed CD8^+^ T cell exhaustion. NR-encased hydrogel system and oncolytic adenovirus loaded with ApoA1 activate anti-tumor immunity through targeting the disordered cholesterol metabolism in GBMs. NOS2, nitric oxide synthase 2; NO, nitric oxide; Arg1, arginase 1; Trp, tryptophan; TDO, tryptophan-2,3-dioxygenase; 3-HA, 3-hydroxyanthranilic acid; 3-HAO, 3-hydroxy-anthranilic acid oxygenase; QPRT, quinolinic acid phosphoribosyl transferase; NHE1, Na/H exchanger isomer 1; OXPHOS, oxidative phosphorylation; sEV, small extracellular vesicles; Glt-1, glutamate transporter type 1; NR4A2, nuclear receptor subfamily 4 group A member 2; SQLE, squalene monooxygenase; ApoA1, apolipoprotein A1; DHCR7, 7-dehydrocholesterol reductase

Given that the main role of macrophages in the TME is predominantly pro-tumorigenic, numerous strategies have been developed to counteract the role of these cells. Broadly speaking, these strategies can be categorized into two groups: reducing the number of macrophages or altering their function in the TME [65, 66]. The former involves macrophage removal and inhibition of macrophage recruitment in the TME. A significant reduction in macrophage number could be achieved by inhibiting the CSF1-CSF1R signaling pathway, which is essential for macrophage development and maturation [67, 68]. Several clinical trials have been conducted with different antibodies and small molecule drugs targeting CSF1R [69, 70]. For example, the small molecule drug PLX3397, although showing favorable anti-tumor effects in patients with advanced tenosynovial giant-cell tumor, presented tolerance in recurrent glioblastoma patients and the six-month progression-free survival was not significantly improved [71, 72]. Furthermore, macrophage accumulation in the TME is usually mediated by monocyte recruitment through the CCL2-CCR2 signaling axis [73]. Therefore, blocking CCL2 signaling has also emerged as a potential target for antitumor therapy [67, 68]. Whereas the latter remodeling of macrophage functions may be more advantageous, by promoting the polarization of M2-type macrophages towards M1-type. For example, Toll-like receptors agonists or PI3K inhibitors can both promote the polarization of M2-type macrophages toward M1-type and exert anti-tumor effects [74–77]. Although these therapeutic strategies targeting macrophages have achieved significant preclinical therapeutic efficacy, there is still a long way to go before they are widely adopted in clinical practice. As mentioned previously, different subtypes of GAMs have distinct metabolic profiles, and thus targeting macrophage metabolism may become a proven therapeutic tool for gliomas. Here we summarize the currently reported therapeutic modalities for targeting metabolism between GAMs and Gliomas (Fig. 5).

### Targeting amino acid metabolism

GAMs have remarkable plasticity and therefore one of the main strategies to treat gliomas through targeting GAMs metabolism is to foster the conversion of macrophages from M2 phenotype to M1 phenotype, thus reversing the anti-inflammatory TME and promoting anti-tumor immune activation. Arginine is a semi-essential amino acid that can be synthesized in the intestinal renal axis catalyzed by arginine succinate synthase (ASS1) and arginine succinate lyase (ASL), or locally in cells expressing ASS1 and ASL. Proliferatively active cells, such as tumor cells, are typically highly arginine-dependent, but ASS1 expression is deficient in many tumor cells, and when they are deprived of arginine, these cancer cells often undergo disruption of energy metabolism leading to death because they cannot synthesize it themselves, whereas in normal cells, metabolism is not affected by arginine deprivation. Therefore, therapeutic arginine deprivation can starve tumor cells to achieve favorable antitumor effects and has become a potential anti-tumor strategy for several tumor types, including ASS1-negative GBM [78–81]. Although arginine consumer pegylated arginine deiminase (ADI-PEG20) has shown significant efficacy in vivo, in vitro, and in phase I clinical trials in ASS1-expressing deficient GBM, it had no significant effect on ASS1-positive GBM. On this basis, Nabil et al. investigated a therapeutic arginine deprivation strategy for ASS1-positive GBM [82]. Both in vivo and in vitro, ADI-PEG20 combined with radiotherapy was shown to achieve tumor elimination and significantly improved survival rate. Mechanistically, ADI-PEG20 decomposes arginine, recycles citrulline, and induces upregulation of iNOS expression to promote NO production, which interacts with superoxide anion free radicals generated in the target area of radiotherapy, thereby destroying tumor cells. In addition, ADI-PEG20-induced arginine deprivation boosts CD8^+^ T cell infiltration in TME, reduces the number of Foxp3^+^ Treg cells, and improves the immune microenvironment [83]. Meanwhile, this combination treatment also induced the transformation of GAMs into M1 phenotype, enhanced the phagocytic ability of GAMs, and promoted the activation of immune function **(**Fig. 5a**)**. Collectively, arginine deprivation therapy combined with radiotherapy is expected to be an attractive research orientation for glioma treatment in the future

Quinolinic acid, a degradation product of tryptophan, is one of the precursors of NAD^+^, which is an essential cofactor for a variety of enzymes and contributes to the activity of enzymes such as the DNA repair protein PARP [84]. Fellx et al. found that tryptophan was converted to 3-hydroxyanthranilic acid (3-HA) by the action of tryptophan-2,3-dioxygenase (TDO), which produces kynurenic acid. Further, in recurrent gliomas, 3-hydroxyanthranilic acid oxygenase (3-HAO), which is expressed only in microglia, promotes the transformation of 3-HA into quinolinic acid. Under oxidative stress such as irradiation, NAD^+^ is in a depleted state in glioma cells. At this time, the expression of quinolinic acid phosphoribosyltransferase (QPRT), the key enzyme that converts quinolinic acid into NAD^+^, is upregulated as the means of NAD^+^ salvage pathway to promote tumor cell survival and treatment resistance. And QPRT is highly expressed only in glioma cells [85]. Thus, endogenous tryptophan metabolites and the NAD^+^ precursor quinolinic acid render gliomas resistant to oxidative stress and thus acquire resistance to radiotherapy and chemotherapy. Therapeutic resistance in gliomas can be alleviated by depleting intracellular NAD^+^, such as by applying alkylating agents or directly inhibiting NAD^+^ synthesis. In addition, targeting QPRT or the quinolinic acid production pathway in microglia is also a potential therapeutic strategy for glioma **(**Fig. 5b**)**.

### Targeting glucose metabolism

Apart from inducing M1-type polarization of macrophages, altering the tumor immune microenvironment by regulating the metabolic pathways of macrophages, thereby enhancing immune cell function and promoting immune cell infiltration, is also one of the important strategies for glioma treatment. As mentioned earlier, NHE1 is instrumental in maintaining normal pH of tumor cells, keeping the driving force of tumor glycolysis, and forming acidic immunosuppressive TME. Hasan et al. discovered in murine glioma models that temozolomide combined with HOE642, a specific inhibitor of NHE1, significantly promoted infiltration of GAMs and T cells, facilitated Th1 activation, and upregulated mitochondrial oxidative phosphorylation pathway genes in GAMs, enhanced glucose uptake and mitochondrial activity in immune cells, and diminished aerobic glycolysis in GAMs [60]. In NHE1 knockout murine glioma model, it was observed that the number of Treg was reduced, the PD-1 expression of T cells was increased, the sensitivity to temozolomide combined with anti-PD-1 therapy was markedly increased, and the median survival time was prolonged, further demonstrating that NHE1 could be an effective therapeutic target for glioma **(**Fig. 5c**)**.

### Targeting microglia-derived small extracellular vesicles

Extracellular vesicles are important intermediates for information transmission between cells, enabling intercellular communication by carrying loads of different substances. Carmela et al. investigated the effects of microglia-derived small extracellular vesicles (sEV) on glioma cells in vitro and in a C57BL/6N mouse model. They revealed that sEV carrying miR-124 altered the metabolism of glioma cells and reduced the release of lactate, NO and glutamate (Glu), resulting in a significant reduction in tumor size and prolonged survival of mice. In addition, sEV impacted Glu homeostasis and increased the expression of Glu transporter Glt-1 in astrocytes **(**Fig. 5d**)**. The use of sEVs in glioma treatment holds potential due to several advantages. First, sEVs are inanimate substances that can evade clearance by immune system. Second, sEVs can remain stable in vitro for up to several months. Finally, sEVs can be easily produced and stored on a large scale [86].

### Targeting lipid metabolism

Targeting lipid metabolic pathways is also a potential therapeutic strategy for gliomas (Fig. 5e). It has been demonstrated that the high expression of oxidative-stress-responsive genes is associated with a worse prognosis in glioma patients and promotes macrophage polarization towards M2 type in the TME [87]. Ye et al. found that microglia in the tumor tissues of GBM patients were under severe oxidative stress. The nuclear receptor subfamily 4 group A member 2 (NR4A2) acted as a transcription factor to regulate the expression of squalene monooxygenase (SQLE), a key enzyme in cholesterol anabolism, and the aberrant expression of SQLE led to abnormal lipid metabolism in microglia, thus promoting immunosuppression and tumor growth. Therefore, targeting inhibition of NR4A2 or SQLE suppressed tumor growth and reversed CD8^+^ T cell exhaustion [88].

Dysregulated cholesterol metabolism in the glioblastoma microenvironment also promotes tumor progression and immunosuppression. Therefore, targeting the disordered cholesterol metabolism in GBM, Wang et al. developed an oncolytic adenovirus loaded with the cholesterol reverse transporter apolipoprotein A1 (ApoA1), for the treatment of GBM, which restores macrophages phagocytosis by manipulating cholesterol efflux and reactivates anti-tumor immunity [89]. Dong et al. found that in GBM, macrophages highly expressed 7-dehydrocholesterol reductase (DHCR7), increased cholesterol biosynthesis, and served as “cholesterol factory” supplying cholesterol for GBM growth. Therefore, they developed an intracavitary sprayable nanoregulator (NR)-encased hydrogel system to modulate cholesterol metabolism of macrophages. The NR-mediated DHCR7 ablation in macrophages effectively inhibited cholesterol supply and activated T-cell immunity [90].

## Conclusion

Glioma is the most common brain tumor and the current standard treatment is surgical resection combined with postoperative radiotherapy and chemotherapy, but the survival time of patients is still far from satisfactory. Metabolic reprogramming is a hallmark of cancer, targeted metabolic therapy is promising to improve the outcome of glioma patients. Microglia and macrophages infiltrate most extensively in gliomas and are expected to be potential targets for glioma treatment. In this review, we describe the physiological metabolism of GAMs and their metabolic profiles in glioma TME, and conclude the therapeutic schemes for metabolic targets of GAMs. Currently, the main therapeutic strategies include polarization of GAMs to M1 phenotype by regulating metabolism, altering the immunosuppressive TME to provide a favorable condition for immune cell infiltration, or directly inhibiting key enzymes in the metabolic pathway between microglia and tumor cells, thereby suppressing tumor growth. However, there are still some issues to be considered when developing therapeutic strategies and translating into clinical practice for targeting metabolism between GAMs and gliomas.

Firstly, the tumor microenvironment consists of numerous different types of cells with distinct functions, including immune cells and stromal cells. Different types of cells collaborate with each other and work together in the disease progression [91]. Therefore, single-targeting GAMs is difficult to sufficiently alter the tumor microenvironment so as to achieve tumor eradication, and the therapeutic effect may be suboptimal. Secondly, glucose metabolism, lipid metabolism and amino acid metabolism, as the three basic metabolic pathways for cellular energy supply, have an extremely high degree of commonality and universality [92]. It is challenging to achieve individual targeting of a particular metabolic pathway of GAMs. Thirdly, the complexity of metabolism is also manifested in the fact that multiple metabolites can be transformed into each other [93, 94]. When targeting only a single metabolic pathway, the efficacy may be poor, and combination therapy could be an effective tool to improve the efficacy. Finally, the animal models used in preclinical studies are vastly different from humans and cannot fully simulate and reflect the real disease conditions and immune profile of the human body. Hence, the efficacy of drugs that have achieved excellent therapeutic effects in preclinical studies remains to be evaluated in different individual patients.

Preliminary results of therapeutic studies targeting the metabolism of GAMs have been reported, but are mainly limited to preclinical studies, and more efforts are warranted to translate into clinical practice. In conclusion, targeting metabolic reprogramming of GAMs in the glioma microenvironment holds potential in improving treatment efficacy for glioma, further studies are needed to bridge the gap between bench and bedside, and bring survival benefits to glioma patients.

## Data Availability

The data that support the findings of this study are available from the corresponding authors upon reasonable request.

## Abbreviations

TME: Tumor microenvironment
GAMs: Glioma-associated macrophages
CNS: Central nervous system
WHO: World Health Organization
Treg: Regulatory T lymphocytes
LPS: Lipopolysaccharide
NADPH: Nicotinamide adenine phosphate dinucleotide
ROS: Reactive oxygen species
iNOS: Inducible nitric oxide synthase
NO: Nitric oxide
Arg1: Arginase 1
HIF: Hypoxia-inducible factor
VEGF: Vascular endothelial growth factor
DCs: Dendritic cells
NHE1: Na/H exchanger isomer 1
LDH: Lactate dehydrogenase
G6PD: Glucose-6-phosphate dehydrogenase
GPD2: Glycerol-3-phosphate dehydrogenase
PGK1: Phosphoglycerate kinase 1
PDPK1: 3-Phosphatidylinositol-dependent protein kinase 1
ASS1: Arginine succinate synthase
ASL: Arginine succinate lyase
3-HA: 3-Hydroxyanthranilic acid
TDO: Tryptophan-2,3-dioxygenase
3HAO: 3-Hydroxyanthranilic acid oxygenase
QPRT: Quinolinic acid phosphoribosyltransferase
sEV: Small extracellular vesicles
Glu: Glutamate
NR4A2: Nuclear receptor subfamily 4 group A member 2
SQLE: Squalene monooxygenase
ApoA1: Apolipoprotein A1
DHCR7: 7-Dehydrocholesterol reductase
